# Sudden Death in a Rare Case Due to Tracheo-Innominate Artery Fistula

**DOI:** 10.3390/jcm13237112

**Published:** 2024-11-25

**Authors:** Matteo Antonio Sacco, Saverio Gualtieri, Federico Longhini, Eugenio Garofalo, Andrea Bruni, Maria Cristina Verrina, Stefano Lombardo, Santo Gratteri, Isabella Aquila

**Affiliations:** 1 Institute of Legal Medicine, Department of Medical and Surgical Sciences, “Magna Graecia” University of Catanzaro, 88100 Catanzaro, Italy; matteoantoniosacco@gmail.com (M.A.S.); saveriogualtieri@icloud.com (S.G.); mariacristina.verrina@studenti.unicz.it (M.C.V.); stefano.lombardo@unicz.it (S.L.); gratteri@unicz.it (S.G.); 2 Intensive Care Unit, Department of Medical and Surgical Sciences, “Magna Graecia” University of Catanzaro, 88100 Catanzaro, Italy; flonghini@unicz.it (F.L.); eugenio.garofalo@unicz.it (E.G.); andreabruni@unicz.it (A.B.)

**Keywords:** sudden death, intensive care, tracheostomy, trachea innominate fistula

## Abstract

**Background:** Tracheostomy is an essential procedure in cases of respiratory failure in patients requiring long-term ventilation or showing airway obstruction. Tracheostomy has both immediate and long-term complications. Among these, tracheo-innominate fistula is an emergency that is a rare long-term complication. When it occurs, this event is catastrophic for the patient’s life, as it causes death in a very short time due to hemorrhagic shock. Therefore, it is essential to identify risk factors to prevent these cases. **Methods:** We describe the autopsy findings in a case of death from tracheo-innominate fistula of a patient admitted to the Intensive Care Unit. **Results:** The autopsy demonstrated, in addition to the large fistula, the coexistence of a malformation of the cervical spine with a significant increase in the diameter of the neck. Therefore, we emphasize in this case the importance of evaluating risk factors in subjects with tracheostomy by highlighting the role of anatomy and the size of the neck as potential predictable risks. **Conclusions:** The work retraces through a review the pathogenesis of this rare complication and emphasizes the need for early diagnosis and prevention of the risk of death with specific risk scales.

## 1. Introduction

A tracheostomy procedure involves the creation of a stoma at the skin surface of the anterior neck. This procedure allows for direct access to the trachea to facilitate breathing, particularly in patients who require long-term ventilation or have upper airway obstructions [1]. Post-operative care and management of tracheostomy patients are critical to ensure successful recovery and prevent complications. Frequent assessment of the airway and monitoring for tracheostomy tube displacement are essential in the early post-operative period [2]. Airway intervention may be necessary, especially within the first 24 h, during which the tracheostomy lumen should be suctioned hourly to maintain patency [3]. Additionally, the tracheostomy balloon, which prevents secretions from entering the airway, is typically inflated for about 24 h post-surgery [4]. Proper management protocols, including regular cleaning and humidification of the tracheostomy tube, help prevent infections and ensure patient comfort [5]. The tracheostomy procedure is not free from complications and therefore requires surveillance, as both short- and long-term complications can develop.

Immediate post-operative complications of tracheostomy, though infrequent, can be quite severe and require vigilant monitoring [6]. These complications include bleeding, which is the most common and can occur in about 5.7% of cases [7]. Additionally, pneumothorax and pneumomediastinum may develop, leading to respiratory distress and requiring prompt intervention. Other potential issues include airway fire and posterior tracheal wall perforation with subsequent esophageal injury [7]. Long-term complications of tracheostomy can significantly impact a patient’s quality of life and require ongoing management. Tracheal stenosis is the most prevalent serious late complication, characterized by the abnormal narrowing of the tracheal lumen due to fibrosis, often requiring surgical intervention or stenting to maintain airway patency [8]. Other long-term issues include swallowing disorders, which may lead to aspiration and nutritional deficiencies, and voice complaints due to changes in the anatomy and function of the vocal cords [9]. Additionally, fistulas and poor healing can present persistent challenges, requiring regular follow-ups and potential corrective procedures [10,11].

Tracheo-innominate artery fistula (TIF) is a rare but life-threatening complication that can occur after a tracheostomy. This condition is associated with sudden, massive hemorrhage and carries a high mortality rate if not promptly treated [12]. The reported incidence of TIF is relatively low, ranging from 0.1% to 1% following surgical tracheostomy procedures [13]. The condition often manifests as severe bleeding from the tracheostomy site, which can rapidly become fatal if not managed immediately [14]. Due to its rarity, very few works have described this complication with its presentation from an autopsy point of view. In this paper, we present a case of sudden death related to the development of tracheo-innominate fistula. In particular, we emphasize how the autopsy allowed us to evaluate risk factors in the specific case that should be considered and evaluated in the case of patients with tracheostomy with specific risk ranges.

## 2. Case Report

A 34-year-old man with cognitive impairment was admitted to the hospital for hypoxemic Acute Respiratory Failure (ARF) due to pneumonia caused by Mycoplasma pneumoniae (antimicrobial therapy with levofloxacin was initiated). Initially, the patient was admitted to the pulmonology department and treated with high-flow nasal cannula oxygen therapy alternated with non-invasive ventilation.

After seven days, the patient’s condition deteriorated, necessitating admission to the Intensive Care Unit (ICU). Analgosedation with propofol and remifentanil, along with invasive mechanical ventilation, were instituted. Given the severity of the ARF (arterial partial pressure to inspired oxygen fraction [PaO_2_/FiO_2_] of 72 mmHg), the patient was immediately transferred to our academic hospital for potential veno-venous extracorporeal membrane oxygenation (vv-ECMO). Hemodynamic support with low-dose norepinephrine (up to 0.4 mcg/kg/min) was required. However, vv-ECMO was not performed because gas exchange improved after prone positioning (PaO_2_/FiO_2_ = 184 mmHg), a recruiting maneuver, and adjustments to mechanical ventilation settings guided by respiratory mechanics and Electrical Impedance Tomography.

Sixteen-hour prone positioning sessions were carried out from Day 1 to Day 3 after ICU admission. By Day 5, the patient’s respiratory condition had improved, and prone positioning was no longer required. The weaning process was initiated: propofol was discontinued, dexmedetomidine was started and assisted mechanical ventilation was instituted. The patient was arousable and conscious. However, due to an ineffective cough and abundant secretions, a percutaneous tracheostomy was performed on Day 8 without complications. A bronchoscopy with bronchoalveolar lavage was performed, and Staphylococcus aureus was isolated. Based on antibiogram results and minimum inhibitory concentrations (MIC), vancomycin therapy was initiated on Day 10.

In the following days, the patient’s clinical condition progressively improved. However, on Day 18, after an episode of coughing, the patient developed sudden and severe hemoptysis. Bronchoaspiration was performed, and a bronchoscopy revealed a bloody bronchial tree. The bleeding source was not found in the deep airways but was traced to the tracheostomy site. The patient rapidly developed hemorrhagic shock, prompting treatment with tranexamic acid and coagulation factors. An emergency consultation with Ear–Nose–Throat (ENT) specialists led to the decision for urgent surgery. During this time, blood transfusions were administered.

The ENT team performed an immediate surgical incision at the base of the neck, identifying a lesion at the brachiocephalic artery. Three stitches were applied to the damaged artery, but controlling the bleeding proved challenging due to the size of the lesion and the rapid hemorrhage. The hemorrhagic shock worsened, leading to cardiac arrest. Advanced Cardiac Life Support (ACLS) maneuvers were initiated, and after ten minutes of unsuccessful attempts at return of spontaneous circulation, veno-arterial ECMO was attempted. However, the hemorrhagic shock was so severe that femoral vein cannulation was unsuccessful. After 40 min of resuscitation attempts and no return of spontaneous circulation, the patient was pronounced dead. A diagnostic confirmation was subsequently requested.

### Autopsy Findings

**Figure 1:**
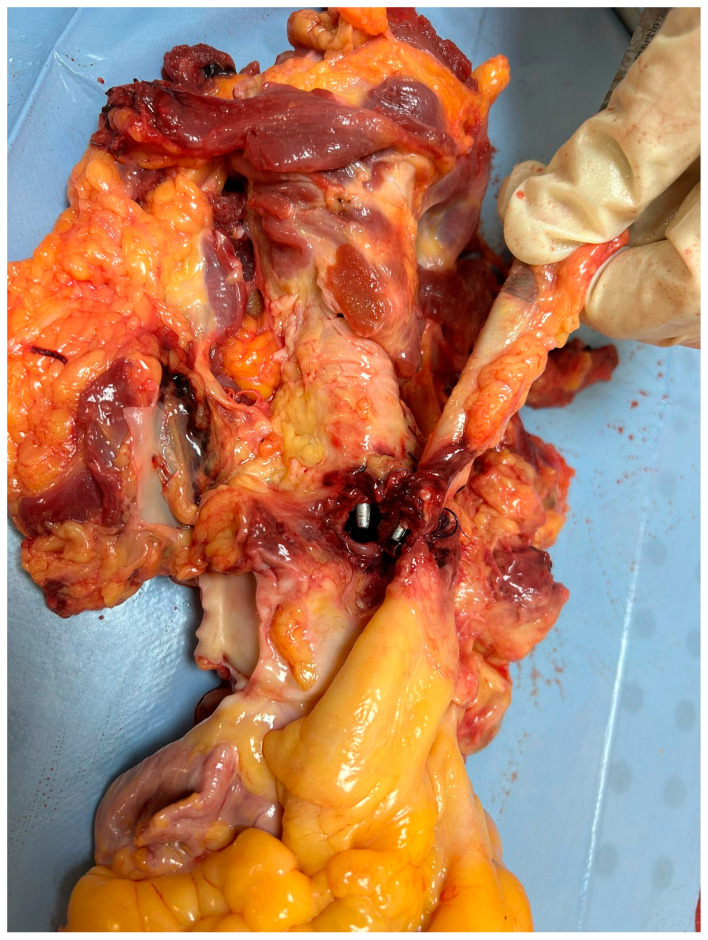
Analysis of the connection between trachea and artery.

**Figure 2:**
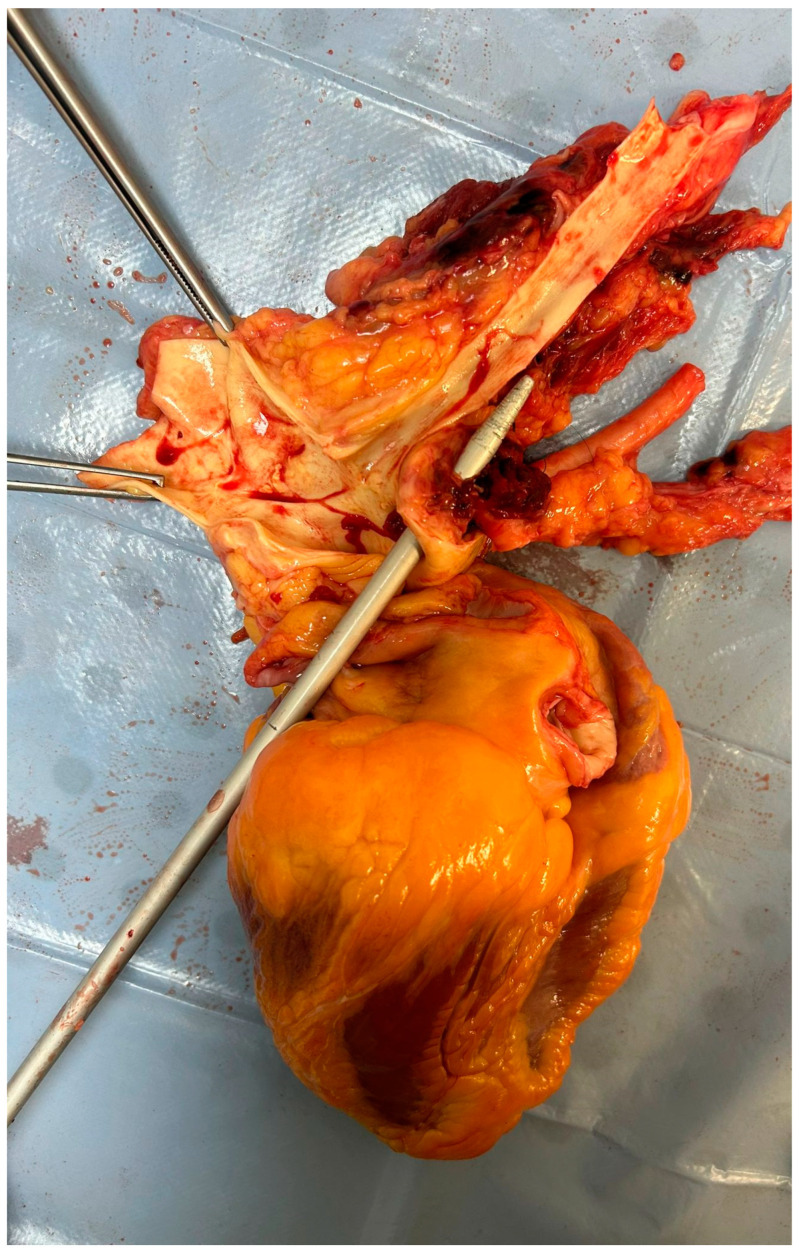
Visualization of the right brachiocephalic artery injury.

**Figure 3:**
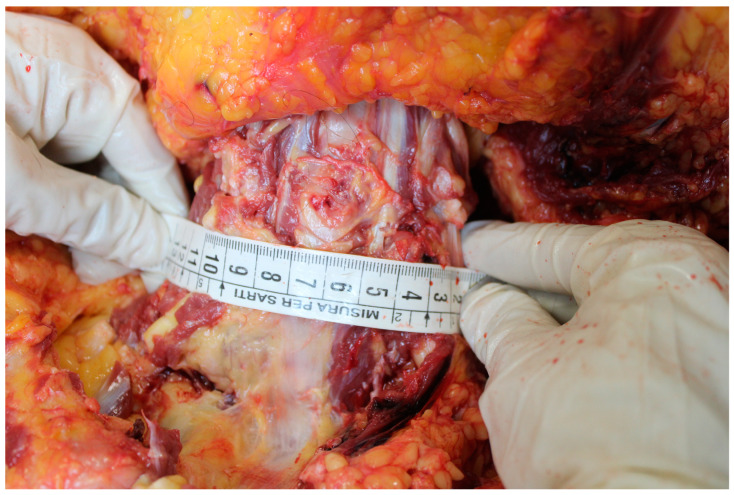
Measuring the depth of the neck.

**Figure 4:**
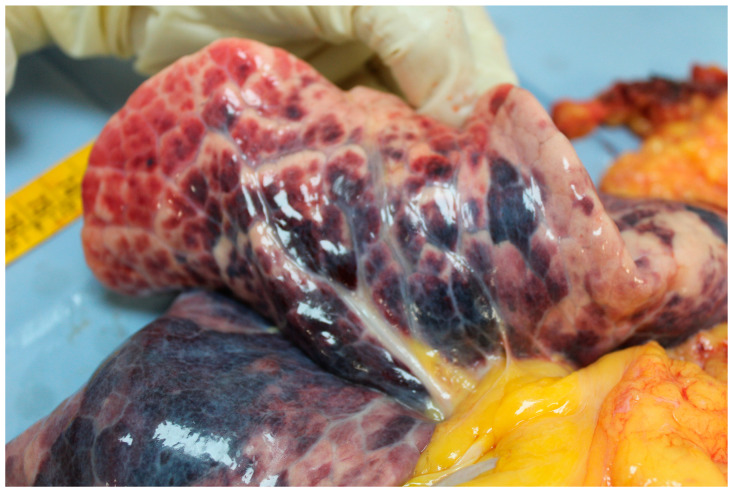
Signs of inhaling blood into the lungs.

The autopsy showed on external examination the presence of a surgical incision at the base of the neck. The section documented a malformation at the cervical level with an increase in the circumference of the cervical spine greater than normal (width equal to approximately 12 cm). A fistula was then identified with the use of a special probe between the trachea and the right brachiocephalic trunk with a breach of approximately 0.5 cm in diameter while the remaining part of the lesion was sutured. The lungs appeared hyper-expanded with polka-dotted areas determined by the inhalation of blood into the airways (Figure 1, Figure 2, Figure 3 and Figure 4). Microscopic analysis of local tissues around the tracheostomy revealed thinning of the tracheal wall, loss of cartilage and infiltration of inflammatory cells, such as neutrophils and macrophages. Pulmonary tissue showed signs of secondary damage with hemorrhagic areas, caused by acute blood loss from the fistula.

## 3. Materials and Methods

An autopsy was performed with an external examination, measurement of external lesions and subsequent analysis of the head, neck, thorax and abdomen. The tongue–trachea–heart–lung block was extracted. Analysis of the tracheal lesion involved the use of probes for evaluation via the tracheal and the affected vessel. The individual organs were then weighed and analyzed. The lesions were documented photographically. A literature review was then performed using the Pubmed and Scopus search engines. The following key words were entered: *Tracheo Innominate Artery Fistula and autopsy OR Tracheo Innominate Artery Fistula and death*. Only English-language papers describing cases of death or autopsy data emerging from the analysis of cases with tracheo-innominate artery fistula were included. The timeline included papers published in the last 20 years (2004–2024). Abstracts or non-peer-reviewed sources were not included.

## 4. Results and Discussion

### 4.1. Clinical Presentation and Diagnosis

According to the literature, TIF is a rare but life-threatening complication that most commonly occurs within 7–14 days after the tracheostomy procedure, although cases have been reported outside this window. Studies indicate that the incidence of TIF is estimated to range from 0.1% to 1%, with mortality rates exceeding 75% in untreated cases. For instance, a retrospective analysis by Nishimura et al. highlighted that over 85% of TIF cases occurred within the first two weeks post-tracheostomy, underscoring this period as critical for heightened surveillance [15]. Similarly, a systematic review by O’Malley et al. (2021) identified that factors such as low placement of the tracheostomy tube and excessive cuff pressure contribute to early erosion of the trachea and adjacent vessels, typically manifesting during the early post-operative phase [16]. The majority of TIF cases occur within 7 to 14 days post-tracheostomy. This timeframe reflects the period when erosive processes, such as persistent contact between the tracheostomy tube and the innominate artery, are most active. However, delayed cases, including cases occurring after 20 days or more, have been reported. The clinical presentation of a tracheo-innominate artery fistula (TIF) often involves a sudden and massive hemorrhage, which is a hallmark symptom, making it a medical emergency [17]. Other symptoms might include sentinel bleeding, which is minor bleeding that precedes the major hemorrhage and can serve as an early warning sign [16]. Patients might also experience difficulty breathing or a sensation of choking due to the compromised airway. Given the rarity of the condition, with an incidence of 0.1–1% following surgical tracheostomy, healthcare providers should maintain a high index of suspicion [18]. Early recognition of these symptoms is crucial for prompt intervention and improved patient outcomes [19]. To diagnose a tracheo-innominate artery fistula, several diagnostic tools and techniques are employed. Imaging studies such as computed tomography (CT) angiography can provide detailed visuals of the trachea and surrounding vasculature, helping to identify the presence of a fistula [19]. Bronchoscopy may also be utilized to directly visualize the trachea and locate the source of bleeding. Additionally, endoscopy can be instrumental in ruling out other potential causes of bleeding and airway obstruction [20]. These diagnostic methods are essential for confirming the diagnosis and planning the appropriate surgical intervention, as TIF requires immediate and precise treatment.

In the differential diagnosis of tracheo-innominate artery fistula, it is important to consider other conditions that can present with similar symptoms. These include tracheal or bronchial bleeding from other causes, such as lung cancer or bronchiectasis [21]. Additionally, conditions like esophageal varices or gastric ulcers might also cause significant bleeding and should be ruled out [21]. Understanding the potential differential diagnoses is critical to avoid misdiagnosis and ensure that patients receive appropriate and timely treatment [22].

### 4.2. Treatment Options and Management

The primary approach involves the resection of the innominate artery, followed by creating an interposition vein graft, typically using a saphenous or jugular vein, secured with a running 5-0 Prolene suture [23]. This procedure not only halts the immediate bleeding but also aims to restore vascular integrity and maintain adequate blood flow. In some cases, endovascular stent grafting has been employed as a less invasive alternative, showing promising outcomes in terms of patient survival [16]. Prompt surgical intervention is vital, as the mortality rate without operative measures approaches 100% [15]. Non-surgical management strategies for TIF are generally limited and often serve as temporary measures until surgical intervention can be performed. Key non-surgical approaches include airway management through endotracheal intubation and the use of wall suction to remove blood from the oropharynx and trachea, ensuring that the airway remains clear [24]. Despite these measures, the prognosis for patients who do not undergo surgery is grim, with death being almost inevitable. The lack of substantial evidence supporting imaging or other non-invasive diagnostic tools further complicates the non-surgical management of TIF. As such, non-surgical strategies are typically seen as interim solutions rather than definitive treatments.

Interventional radiology (IR) plays a critical role in both diagnosing and managing tracheo-innominate fistula (TIF), particularly in hemodynamically unstable patients. Digital subtraction angiography (DSA) can accurately localize the bleeding source when other diagnostic modalities, such as endoscopy, are not feasible. In cases of severe instability, rapid localization of vascular defects using endovascular catheterization enables targeted therapeutic interventions. A case reported by Khanafer et al. demonstrated the successful use of DSA to identify extravasation from the innominate artery into the trachea in a patient with acute bleeding, followed by effective placement of a stent graft [23].

Therapeutically, IR offers minimally invasive options like endovascular stenting and embolization. Endovascular stent grafts, in particular, have shown promise as either definitive or bridging therapies. Taechariyakul et al. conducted a meta-analysis highlighting lower mortality (9% vs. 23%) and complication rates (30% vs. 50%) with endovascular interventions compared to traditional surgery in managing arterial fistulas [25,26].

This approach is especially useful in patients unfit for immediate open surgery, offering temporary control of hemorrhage while stabilizing the patient for subsequent definitive treatment.

While the endovascular approach has advantages, it is not without risks. Complications such as graft infections, rebleeding due to erosion and ischemic events from vessel occlusion remain significant considerations. For example, improper positioning of stent grafts can lead to cerebral ischemia or compromised blood flow to the subclavian or carotid arteries. Despite these limitations, case studies consistently underscore the utility of IR techniques in reducing mortality and providing life-saving interventions in emergencies.

Post-operative care and the management of complications are crucial components of the overall treatment plan for patients with TIF. Following surgical repair, patients require close monitoring for any signs of complications, such as infection, graft failure, or recurrent hemorrhage [23]. The success of the surgical intervention depends significantly on the quality of post-operative care, which includes vigilant observation, appropriate antibiotic therapy and regular follow-up assessments. Although the primary surgical repair addresses the immediate life-threatening issue, the potential for post-operative complications remains high, necessitating comprehensive care to ensure long-term patient survival and recovery.

### 4.3. Prognosis and Mortality

Survival rates for TIFs are alarmingly low, primarily due to their association with massive hemorrhage and high mortality [1]. Key factors influencing prognosis include the speed of diagnosis, immediate control of bleeding and the urgency of surgical intervention [16]. Without prompt and definitive management, the mortality rate can reach nearly 100%, making rapid response crucial for patient survival [19]. The highest risk period for TIFs typically occurs within the first three weeks post-tracheostomy, underscoring the importance of vigilant monitoring during this timeframe [19]. Effective management strategies must prioritize maintaining a patent airway while simultaneously controlling hemorrhage to improve outcomes [20].

Case studies and statistical data further highlight the deadly nature of TIFs. The incidence rate after surgical tracheostomy ranges from 0.1% to 1%, with a peak occurrence between 7 and 14 days post-procedure [23]. Literature review searches have been conducted to summarize mortality associations, revealing that untreated tracheoarterial erosion has a 100% mortality rate [27]. For example, a study summarized by Ward et al. in a systematic review indicated that nearly all patients succumb to this condition without timely surgical intervention [27]. Preventive measures and future research directions are essential in addressing the high mortality rates associated with TIFs. Current research predominantly focuses on management strategies rather than prevention [24]. Fundamental preventive measures may include procedures such as innominate artery ligation and the use of vascular grafts to reduce the risk of TIF formation [16,24]. Additionally, developing a simple algorithm for the management of TIFs can help control bleeding in a significant number of cases, providing a structured approach to handling this life-threatening complication [2]. Future research should aim to explore more preventive strategies and improve the overall understanding of TIF pathogenesis and management.

### 4.4. Evaluation of Risk Factors and Prevention

Several common causes and risk factors contribute to the development of tracheo-innominate artery fistula. One major risk factor is the prolonged or improper positioning of the tracheostomy tube below the fourth tracheal ring, which increases mechanical pressure on the trachea and adjacent vascular structures [5]. Additionally, the use of corticosteroids and radiation therapy to the neck area can compromise tissue integrity, making it more susceptible to erosion and fistula formation [1]. Recurrent episodes of hypotension, which cause ischemia, also play a role in increasing the risk of this life-threatening complication [1]. Together, these factors create an environment conducive to the erosion of the tracheal wall and the adjacent innominate artery, leading to fistula formation [6]. The pathophysiology of tracheo-innominate artery fistula primarily involves mechanical and ischemic damage to the trachea and innominate artery. Overinflation of the tracheostomy tube cuff can exert excessive pressure on the posterior aspect of the innominate artery, causing erosion and subsequent fistula formation [7]. This pressure-induced damage disrupts the integrity of both the tracheal wall and the arterial wall, creating an abnormal connection between the two structures [6]. The continuous mechanical irritation and ischemia exacerbate the erosion process, ultimately leading to the formation of the fistula. Understanding these mechanisms is crucial for preventing and managing this severe complication [1].

In our case, we emphasize as a further risk factor the presence of a deformation of the cervical spine, characterized by width and anterior–posterior dimensions (depth) significantly greater than normal, which may have facilitated the contact of the tracheal tube with the wall and therefore the formation of the fistula. In fact, the dimensions of the cervical spine were significantly larger in diameter and depth than normal, and due to the particular anatomy of the neck, the operator was forced to position the tube lateralized to the right on the tracheal wall. We therefore believe it is essential to pay attention to the anatomical characteristics and dimensions of the neck and cervical spine as a potential risk factor in these cases and an indicator of active vigilance. Furthermore, we point out that, to date, there are no known risk scales for tracheo-innominate fistula, and we emphasize the need for them to be created with a preventive calculation of the risk of complications.

## Data Availability

Data are contained within the article.
